# Tuberculosis: Role of Nuclear Medicine and Molecular Imaging With Potential Impact of Neutrophil-Specific Tracers

**DOI:** 10.3389/fmed.2021.758636

**Published:** 2021-12-10

**Authors:** Stuart More, Mohlopheni J. Marakalala, Michael Sathekge

**Affiliations:** ^1^Division of Nuclear Medicine, Department of Radiation Medicine, University of Cape Town, Cape Town, South Africa; ^2^Department of Nuclear Medicine, University of Pretoria and Steve Biko Academic Hospital, Pretoria, South Africa; ^3^Nuclear Medicine Research Infrastructure, Steve Biko Academic Hospital, Pretoria, South Africa; ^4^Africa Health Research Institute, Durban, South Africa; ^5^Division of Infection and Immunity, University College London, London, United Kingdom; ^6^School of Laboratory Medicine and Medical Sciences, College of Health Sciences, University of KwaZulu-Natal, Durban, South Africa; ^7^Division of Immunology, Department of Pathology, University of Cape Town, Cape Town, South Africa

**Keywords:** tuberculosis, formyl peptide receptor, Gallium-68, cFLFLF, nuclear medicine, molecular imaging, PET/CT

## Abstract

With Tuberculosis (TB) affecting millions of people worldwide, novel imaging modalities and tools, particularly nuclear medicine and molecular imaging, have grown with greater interest to assess the biology of the tuberculous granuloma and evolution thereof. Much early work has been performed at the pre-clinical level using gamma single photon emission computed tomography (SPECT) agents exploiting certain characteristics of *Mycobacterium tuberculosis* (*MTb*). Both antituberculous SPECT and positron emission tomography (PET) agents have been utilised to characterise *MTb*. Other PET tracers have been utilised to help to characterise the biology of *MTb* (including Gallium-68-labelled radiopharmaceuticals). Of all the tracers, 2-[^18^F]FDG has been studied extensively over the last two decades in many aspects of the treatment paradigm of TB: at diagnosis, staging, response assessment, restaging, and in potentially predicting the outcome of patients with latent TB infection. Its lower specificity in being able to distinguish different inflammatory cell types in the granuloma has garnered interest in reviewing more specific agents that can portend prognostic implications in the management of *MTb*. With the neutrophil being a cell type that portends this poorer prognosis, imaging this cell type may be able to answer more accurately questions relating to the tuberculous granuloma transmissivity and may help in characterising patients who may be at risk of developing active TB. The formyl peptide receptor 1(FPR1) expressed by neutrophils is a key marker in this process and is a potential target to characterise these areas. The pre-clinical work regarding the role of radiolabelled N-cinnamoyl –F-(D) L – F – (D) –L F (cFLFLF) (which is an antagonist for FPR1) using Technetium 99m-labelled conjugates and more recently radiolabelled with Gallium-68 and Copper 64 is discussed. It is the hope that further work with this tracer may accelerate its potential to be utilised in responding to many of the current diagnostic dilemmas and challenges in TB management, thereby making the tracer a translatable option in routine clinical care.

## Introduction

Although advances in anatomical imaging of tuberculosis (TB) cannot completely characterise the natural course of this disease, nuclear medicine and molecular imaging, particularly positron emission tomography/computed tomography (PET/CT) imaging, has developed rapidly and is now able to provide information on the biology of TB at diagnosis and in assessing treatment response.

From a historical perspective, Gallium-67-citrate ([^67^Ga]Ga-citrate) was one of the first radioisotope workhorses used in the management of TB and had been employed in the clinical practise to give an indication of disease activity, response assessment, and disease extent (1, 2) including the ability to differentiate infection from *Mycobacterium tuberculosis* (*MTb*) from non-*MTb* lesions (3). Work had also been performed in patients coinfected with human immunodeficiency virus (HIV) (4–6).

Thallium-201 chloride[^201^Tl] has been used in some works to distinguish malignant lesions from tuberculous ones (7–9), with further work demonstrating its role in characterising pulmonary and extrapulmonary TB (10–12). Similar early work was performed with Technetium 99m sestamibi (MIBI) ([^99m^Tc]Tc-MIBI) and [^99m^Tc]Tc-tetrofosmin in the management of TB (13–17).

2-deoxy-2-[^18^F]fluoro-D-glucose (2-[^18^F]FDG) has been the most widely studied PET radiopharmaceutical in TB, with a growing interest in Gallium-68-based radiopharmaceuticals. As we anticipate a more specific solution to answer the biology of the granuloma, the neutrophil has shown to play a key role in the pathogenesis of TB, with their presence of neutrophils predicting increased disease severity.

**Figure 1:**
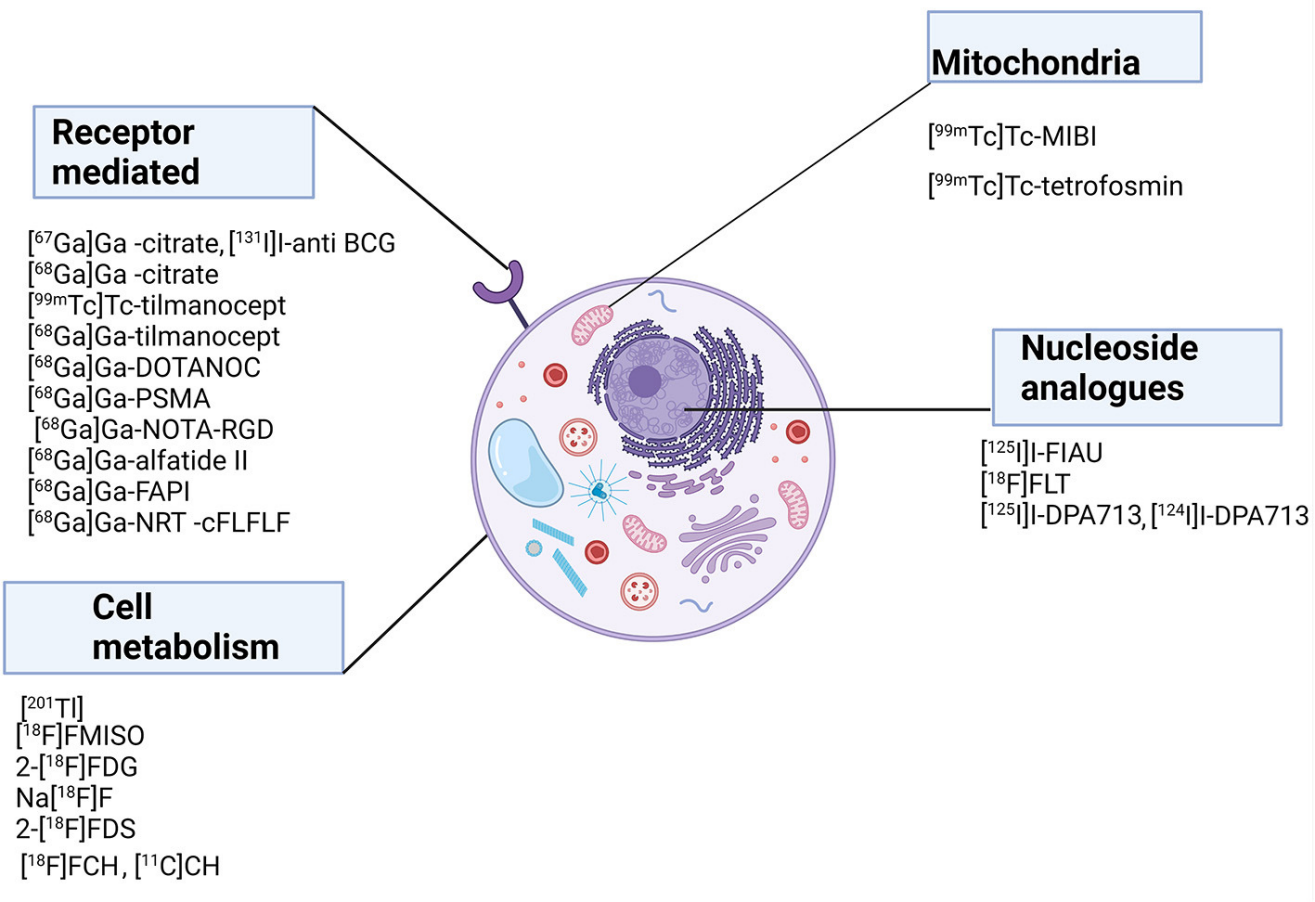
Schematic representation of a host cell and the radiopharmaceuticals listed according to their mechanisms of action.

**Figure 2:**
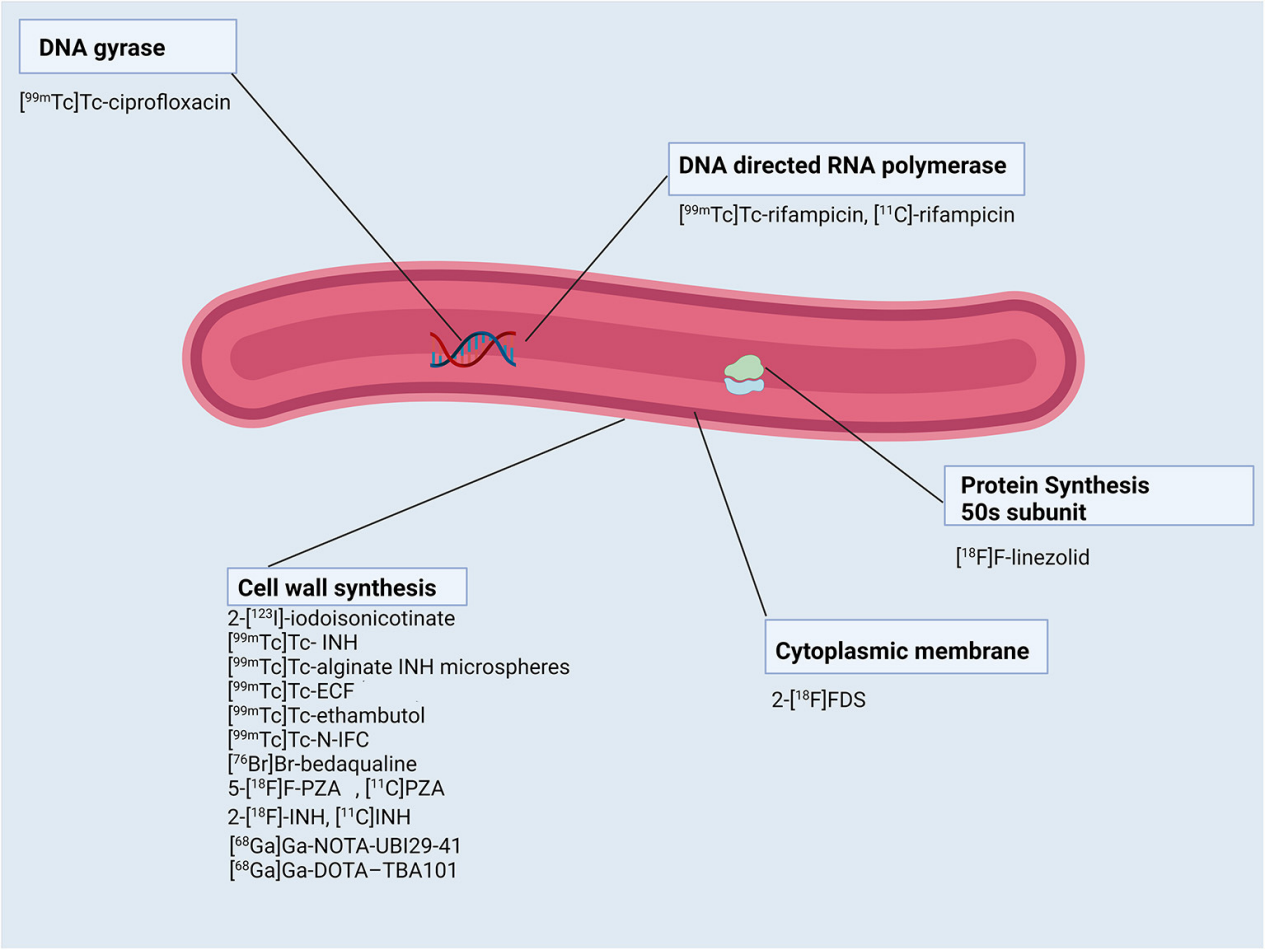
Schematic representation of *Mycobacterium tuberculosis* bacillus and the radiopharmaceuticals listed according to their mechanisms of action.

Figures 1, 2 show a schematic review of the targets of the various radiopharmaceuticals discussed in this review based on their mechanism of action (please also refer to Table 1).

**Table 1:** An overview of the tracers used in imaging and characterising TB (apart from Gallium-68-labelled radiopharmaceuticals).

**Radiotracer**	**Target**	**Stage of investigation**			**Sensitivity and specificity (if clinical data available)**	
		***In vitro***	**Pre-clinical**	**Clinical**	**Sensitivity (%)**	**Specificity (%)**
**Gamma SPECT tracers**						
[67Ga]Ga-citrate	Transferrin receptor, ferritin, lactoferrin	✕	✕	✓ (18–21)	83–100	60–93
[^201^Tl]	Sodium potassium ATPase pump	✕	✕	✓ (9)	88.2	71.4
[^99m^Tc]Tc-MIBI	Mitochondria	✓ (22, 23)	✓ (22, 23)	✓ (22, 23)	86	88
[^99m^Tc]Tc-tetrofosmin	Mitochondria	✓ (22)	✓ (22)	✓ (22)	94	96
[^125^I]I-FIAU	Bacterial TK	✓ (24)	✓ (24)	✕		
[^125^I]I-DPA713	Translocator protein	✕	✓ (25, 26)	✕		
[^131^I]I-anti-BCG	IgM antibody	✓ (27, 28)	✓ (27, 28)	✕		
[^99m^Tc]Tc-tilmanocept	Mannose receptor CD 206	✕	✕	✕		
2-[^123^I]-iodoisonicotinate	Enoyl-ACP reductase	✕	✓ (29)	✕		
[^99m^Tc]Tc-INH	Enoyl-ACP reductase	✓ (30, 31)	✓ (30, 31)	✓ (30)	-	-
[^99m^Tc]Tc-alginate INH	Enoyl-ACP reductase	✓ (32)	✓ (32)	✕		
[^99m^Tc]Tc-N-IFC	Enoyl-ACP reductase	✓ (33)	✓ (33)	✕		
[^99m^Tc]Tc-ECF	Bacterial cell wall	✓ (34)	✓ (34)	✕		
[^99m^Tc]Tc-rifampicin	Beta subunit RNA polymerase	✕	✕	✕		
[^99m^Tc]Tc-Ethambutol	Mycobacterial cell wall	✓ (35, 36)	✓ (35, 36)	✓ (37)	94.9	83.3
[^99m^Tc]Tc-ciprofloxacin	DNA-gyrase	✕	✕	✓ (38)	93	71
**PET tracers**
2-[^18^F]FDG	GLUT	✓ (39)	✓ (40–43)	✓ (43–49)	71.4–100	62.9–100
[^18^F]F-choline [^11^C] choline	Choline transporter	✕	✕	✓ (50, 51)	-	-
[^18^F]fluoro- L-thymidine	TK-1	✓	✓	✓ (52)	68.75	76.92
Na[^18^F]F	TB lesion microcalcification	✓	✓	✕		
[^124^I]I-DPA	Translocator protein	✓ (53)	✓ (53)	✕		
5-[^18^F]fluoropyrazinamide	Bacterial cell wall	✓ (54, 55)	✓ (54)	✕		
2[^18^F]-INH, [^11^C]INH	Enoyl-ACP reductase	✓ (56)	✓ (56)	✕		
2-[^18^F]FDS	Cytoplasmic membrane bacteria	✓ (57)	✓ (57)	✕		
[^11^C]C-rifampicin	beta subunit RNA polymerase	✓ (58, 59)	✓ (58, 59)	✓ (58, 59)	-	-
[^18^F]F-linezolid	Bacterial 50S ribosomal subunit	✓ (60)	✓ (60)	✕		
[^76^Br]Br-bedaqualine	mycobacterial ATP synthase	*in vivo* (61)	✕	✕		
[^18^F]FMISO	TB lesion hypoxia	*in vivo* (62)	✕	✓ (62)	-	-

*✓: Indicates work that has been performed in the respective domain*.

*✕: Indicates work that has not been performed in the respective domain*.

*-: Indicates data unavailable*.

It is the hope of this review to look at the current molecular imaging modalities available in mapping out TB and review the potential role a neutrophil-specific tracer may have in the management of TB.

## Methodology

A comprehensive literature search was performed in the PubMed, EbscoHost, Web of Science Scopus, and Cochrane databases to identify articles published up until June 2021 regarding the role of nuclear medicine and molecular imaging in imaging TB, particularly neutrophils and utility of a neutrophil-specific tracer and the potential role of imaging the TB granuloma. Different combinations of search terms (and their various permutations) were used including “neutrophil,” “leucocyte,” “nuclear medicine,” “PET/CT,” “SPECT/CT,” “SPECT,” “^99m^Tc,” “HMPAO,” “Indium111-oxine,” “F18,” “FDG,” “Copper64,” “Gallium-68,” “tuberculosis,” “granuloma,” “FPR1,” and “cFLFLF” “radiolabelled antituberculous treatment.” The references of the resultant articles were also used to identify additional manuscripts. A total of 182 key articles were identified that discussed the spectrum of nuclear medicine and molecular imaging in TB, with the role of neutrophil-specific imaging in this context. These articles form the basis of this manuscript.

## TB IN 2021

Although there have been many major advances in the field of medicine, TB continues to be one of the world's most devastating diseases, resulting in marked morbidity and mortality. According to the WHO Global Report 2020, 10 million (range 9–11 million) people worldwide contracted TB in 2019, with ~1.2 million (range 1.1–1.3) dying of the disease, which included about 2,51,000 patients who also had HIV co-infection (63). More recently, the current prevalence rates of TB in South Africa are estimated at 852 per 1,00,000 in a recent survey conducted by the South African Medical Research Council (64). Diagnostic imaging of TB includes both plain chest radiographs (CXR) and computed tomography (CT) in more resourced areas. Anatomical imaging (including MRI) is limited in detailing the early stages of TB infection, and changes may only be evident once there has been significant tissue damage or alteration thereof, particularly in the later stage of most diseases. They also are limited in distinguishing sterile inflammation from infection or differentiating between different aetiologies of disease (65). PET/CT imaging has begun to make some traction in being able to answer some of these questions, it is, however, still not fully validated yet for routine clinical use.

Culture remains the reference standard for the diagnosis of TB; however, the culture takes between 2 and 8 weeks to grow sensitive bacteria and is dependent on the burden of mycobacteria present in the sample (66). The Xpert MTB/RIF assay in recent times has added to the tools available to diagnose TB, the test rapidly detects DNA of *MTb* nucleic acid present in sputum and simultaneously assesses genetic mutations predicting rifampicin resistance. The specificity is comparable to that of culture at 98% (67). The Xpert MTB/RIF ultra, which utilises the same GeneExpert platform as Xpert MTB/RIF, was recommended by the WHO in 2017 owing to its improved sensitivity (63). The major limitation of these assays is the inability to monitor disease when compared to culture (44). Other methods for the detection of TB have been developed which are still being validated for routine clinical use (68).

Hence, other non-invasive methods have been developed to assist in the diagnosis of *MTb*.

Nuclear medicine and molecular imaging, with its ability to trace pathophysiological processes using radiopharmaceuticals, are seen as a viable option and adjunct in the holistic management of TB.

## Role in Imaging Tb Using Gamma Spect Agents

Molecular imaging has been utilised within the pre-clinical framework and translated into the clinical setting with several imaging probes having been developed to assist in the diagnosis of infection and inflammation. Single photon emission computed tomography (SPECT) molecular probes were developed to assist in the diagnosis and management of TB, aiming to improve the specificity of the detection of *MTb* infection.

One of the first studies evaluated Technetium 99m sestamibi (MIBI) ([^99m^Tc]Tc-MIBI) and ([^99m^Tc]Tc-tetrofosmin) in TB, demonstrating high uptake of the tracers owing to increased mitochondrial content of the epithelioid cells in the granulomatous lesions (22). Increased uptake of [^99m^Tc]Tc-MIBI was also seen in *MTb* cells when compared to myocytes or fibroblast cultures (with peak activity shown at 15 min), paving the way to understand the mechanism of uptake in *MTb* cells (23).

The nucleoside 1-(2′-deoxy-2′-fluoro-b-D-arabinofuranosyl)-5- iodouracil (FIAU) (also known as fialuridine) labelled with iodine-125 has also been utilised as a tracer to assist in invasive detection and localisation of *MTb* (69). FIAU forms part of the nuclear acids used by microorganisms and is a substrate for bacterial thymidine kinase (TK). Phosphorylation by TK allows for trapping within microorganisms. When *MTb* was modified to express bacterial TK, [^125^I]I-FIAU was shown useful to measure mycobacterial load in infected mice (24).

Work related to the role of activated macrophages which express translocator protein on the mitochondrial membrane for lipid transport has been explored, predominantly within the neuropsychiatric spectrum (70, 71). The translocator protein (TSPO) is an 18-kDa trans-mitochondrial membrane channel used for the transport of cholesterol and other endogenous ligands. TSPO is also expressed highest in steroidogenic tissues, heart, lungs, and immune cells such as macrophages. Radiolabelled *N,N*-diethyl-2-[2-(4-methoxyphenyl)-5,7-dimethylpyrazolo[1,5-*a*]pyrimidin-3-yl]acetamide (DPA713) has been used as a target to map out inflammation associated with pulmonary TB and has been shown to preferentially accumulate in macrophages and phagocytic cells (25). When [^125^I]I-DPA713 was compared to 2-[^18^F]FDG in a mouse model, uptake of [^125^I]I-DPA713 was found to be more discrete when compared to 2-[^18^F]FDG which showed more diffuse uptake in tuberculous lesions (25, 26).

In antibody-mediated work, [^131^I]I-anti-BCG antibody was used as a potential target for TB owing to the similarity between *MTb* and *Mycobacterium bovis*. In a pre-clinical rabbit model, it was shown to localise to tuberculous lesions but was also seen in other organs such as the heart, liver, spleen, and kidneys (27). No further significant work has been performed with this agent, besides Lee et al. who used the antibody fragment F(ab)′ against BCG with radiolabelled with ^131^I. The tracer accumulated in tuberculous lesions in rats while being cleared from syphilitic lesions in the same animal model (28).

[^99m^Tc]Tc-tilmanocept, which is a molecular marker for the mannose receptor CD206, has been utilised in lymphoscintigraphy in oncology (72, 73) and also limited phase 1 studies in mapping atherosclerotic inflammation (74) and rheumatoid arthritis (75, 76). Enhanced expression of CD206 in macrophages, particularly in lung and pleural tissue with caseating granuloma, has portended a poorer prognosis in patients with these lesions (77). Therefore, [^99m^Tc]Tc-tilmanocept may be a future marker for mapping the physiology of the granuloma which is currently underway with a PET-labelled tracer, [^68^Ga]Ga-tilmanocept (78).

## Radiolabelled Antituberculous Spect Agents

The rationale of using radiolabelled antituberculous drugs has long been developed and not necessarily translated into clinical care but has given context into understanding the bioavailability and pharmacokinetics of the drugs used in the treatment regimen of TB.

Radiolabelled isoniazid as 2-[^123^I]-iodoisonicotinate was initially pioneered with the potential to assist in differentiating intracranial masses (tuberculoma vs. glioma), and CNS TB in immunosuppressed subjects (29). Owing to the low labelling efficiency (LE), better complexes were developed by Singh et al., showing more than 95% LE and persistent uptake in tubercular lesions in rabbits, with transient uptake seen in non-tuberculous lesions (30, 31). This level of LE was also seen by Roohi et al., whose results were concordant with Singh et al. (79) Samuel et al. (80) performed another pre-clinical study but did not show any clinically significant uptake by *MTb* in the *in vitro* cell-binding studies. Similar work has been demonstrated using [^99m^Tc]Tc – IFC, an isoniazid derivative (33). [^99m^Tc]Tc-alginate INH microspheres were utilised in rabbits, demonstrating 96% LE and showing moderate lung uptake in TB lesions (32). Clinical translation of radiolabelled isoniazid was seen in six patients who had confirmed extrapulmonary TB, demonstrating moderate uptake in these lesions (81). No further work has been seen with gamma agent translation of radiolabelled isoniazid.

Radiosynthesis and biodistribution of [^99m^Tc]Tc-rifampicin has only been seen in the pre-clinical evaluation of MRSA in artificially infected rats and rabbits. Gamma radiolabelled agents have not been translated to mapping TB infection (82). It is important to note that no further investment has been made into the translation of Technetium 99m-based radiopharmaceutical for TB imaging using rifampicin.

Targets using Technetium 99m-labelled ethionamide ([^99m^Tc]Tc-N-IFC) were also explored in the pre-clinical context and as with [^99m^Tc]Tc-rifampicin and have not progressed beyond the pre-clinical stage (34).

[^99m^Tc]Tc-Ethambutol was initially developed for studying renal excretion and brain imaging owing to the similar structure to its derivatives (35). With ethambutol's key mechanism of action being the inhibition of cell wall synthesis of *MTb*, the utility of radiolabelled ethambutol has been investigated to highlight the clinical efficacy of therapy in the management of *MTb*. Recent work has demonstrated a sensitivity and specificity of 94.9 and 83.3%, respectively, for detecting TB at any sites (PTB and EPTB) with microbial culture used as the gold standard (37). Work related to [^99m^Tc]Tc-ethambutol in spinal TB has also been explored (83). Current limitations of this agent include uptake in normal parenchyma (which will preclude visualisation of areas with uptake above background activity), liver excretion (which hampers the assessment of potential abdominal TB), and overall poorer target to background ratio as a result of the former pitfalls described (36).

Other radiolabelled TB drugs such as [^99m^Tc]Tc-ciprofloxacin have been utilised in clinical practice to differentiate between active and inactive TB (84) and assess response to therapy (85) with the limited widespread clinical application (38). More specific probes have subsequently been developed which have limited widespread clinical application of [^99m^Tc]Tc-ciprofloxacin.

Despite radiolabelled leukocytes and antibodies being the standard in infection and inflammation imaging in a variety of conditions (86–88), most of the radiolabelled cells (predominantly neutrophils) may be less sensitive as a gamma agent in imaging TB (89). There have been very few studies demonstrating the use of radiolabelled white cells in imaging TB, with limited scope in translation into the routine clinical space (90–93). Other non-specific agents have been utilised over the last 20 years to map out the pathophysiology of *MTb* and including previously mentioned [^67^Ga]Ga-citrate (18–21), [^99m^Tc]Tc-MIBI, [^99m^Tc]Tc-tetrofosmin, [^201^Tl]Tl-chloride and less utilised tracers such as [^123^I]I-IMP, [^99m^Tc]Tc-DMSA, [^99m^Tc]Tc-citrate, [^99m^Tc]Tc-glucoheptonate, [^111^In]In-octreotide, [^99m^Tc]Tc-MDP, and [^99m^Tc]Tc-ECD (94). With the advent of PET probes, these have very limited utility in the management of TB.

Of the list above, [^67^Ga]Ga-citrate has been the agent with the most breadth of clinical data (3) and can be used in a setting where PET availability is scarce. In addition, the complexity of timing required to complete these studies and the higher radiation burden associated with [^67^Ga]Ga-citrate has made its use less routine.

## Role of Pet Probes in Imaging TB

PET/CT combines the anatomical information obtained from the CT with the functional aspect using a specific radioisotope combined with a pharmaceutical that traces a specific aspect of physiology or pathophysiology.

2-deoxy-2-[^18^F]fluoro-D-glucose (2-[^18^F]FDG) has revolutionised care in oncology owing to its mechanism of action. In the Warburg effect, tumours will consume more glucose than other tissues which is primarily what is exploited in PET/CT imaging using 2-[^18^F]FDG as a tracer (95). Within cancer cells, uptake of 2-[^18^F]FDG is mediated by glucose transporters (GLUT). GLUT1 is the most common of these transporters, with tumours also expressing GLUT3. Once 2-[^18^F]FDG is in the cell, 2-[^18^F]FDG is phosphorylated by hexokinase (HK) giving 2-[^18^F]FDG-6-phosphate which, unlike glucose, cannot be further metabolised owing to its lacking the hydroxyl group (96). Most tumours, in addition, also express HK2, one of the four isoforms of HK. 2-[^18^F]FDG-6-phosphate can only leave the cell by dephosphorylation, which is catalysed by glucose-6-phosphatase.

Inflammatory cells can also express increased levels of GLUT1 and 3 (particularly macrophages, neutrophils, and other inflammatory cells) and increased HK activity (96). Some bacteria have also been found to contribute to 2-[^18^F]FDG uptake by actively taking up the radiotracer (97).

The differential in uptake between inflammatory and normal cells has been shown in tumour tissue by autoradiographic techniques (39). It is this interaction between inflamed tissue when compared to normal cells that is the basis for 2-[^18^F]FDG uptake in TB.

2-[^18^F]FDG PET/CT in the imaging of TB has been widely investigated. 2-[^18^F]FDG has been used to assess TB lesion activity globally, characterise uptake in the lung and mediastinal lymph nodes (45–47), assist in patients by predicting those who may develop TB in latent TB infection (LTBI) (98) identification of subclinical TB, differentiating TB from malignant lesions, differentiating TB from non-tuberculous mycobacterial infections (99, 100), response assessment to TB treatment and also characterise extrapulmonary TB (44, 48, 49, 98).

## Non-Fdg Pet Tracers

Other PET tracers have been used in the imaging of TB to trace different aspects of the pathophysiology of *MTb*. Choline-based derivatives can image the utilisation of choline in the wall of *MTb* (which is composed of many complex lipids) (50, 51). 3′-deoxy-3′-([^18^F]fluoro)-fluorothymidine [[^18^F]fluoro- L-thymidine (FLT)] can be used as a surrogate for cell proliferation as thymidine incorporates into the DNA of the bacterium. [^18^F]FLT has been evaluated in distinguishing malignant from benign lesions in conjunction with 2-[^18^F]FDG (52, 101). [^18^F]F-sodium fluoride (Na[^18^F]F) has only been used in the murine model but has the potential for use in detecting chronic TB from acute infection (102). [^124^I]I-DPA713 was utilised in a paediatric rabbit model of tuberculous meningitis to show its ability to detect *MTb* where it was localised to the activated microglia/macrophages around the TB lesion. These findings were confirmed with immunohistochemical techniques (53).

2-deoxy-2-[^18^F]fluoro-d-sorbitol (2-[^18^F]FDS) is a tracer that was initially designed for tumour imaging but found to have limited use owing to the low accumulation of the tracer *in vitro* (103). 2-[^18^F] FDS has been shown to have pathogen-specific uptake by bacteria and has the ability to distinguish bacterial infection from sterile inflammation (104). The application of 2-[^18^F]FDS in TB may be limited owing to the lack of gene expression of sorbitol subunits in the mycobacteria (105). Experiments have been performed with mycobacteria at the pre-clinical level with no uptake of 2-[^18^F]FDS seen in *MTb* (57).

Despite the efforts of aiming to characterise TB with these non-FDG PET tracers, much more work is required before they can be translated to clinical care.

## Radiolabelled Antituberculosis Pet Agents

Both pyrazinamide and isoniazid have been radiolabelled with PET agents but have yet to have further clinical translation because of their pharmacokinetic properties (54–56).

Radiolabelled rifampicin with PET has shown some promise, with pre-clinical work in mice determining the pharmacokinetics of Carbon 11-rifampicin ([^11^C]-rifampicin) in *MTb*-infected mice. This work showed lower concentrations of tracer in infected necrotic lung specimens when compared to healthy tissue (58). More recently, [^11^C]-rifampicin was used in a first in human trial to map out the antimicrobial concentration time profiles of rifampicin in subjects who were proven with rifampicin-sensitive pulmonary TB. [^11^C]-rifampicin had spatially compartmentalised rifampicin exposure in pathological distant TB lesions, with uptake lowest in the wall of tuberculous lesions and cavities, which may have implications for the development of future host-directed therapies (59). PET tracers such as [^18^F]F-linezolid (60) and Bromine-76 bedaqualine ([^76^Br]Br-bedaqualine) (61) have also been utilised in the pre-clinical setting in murine models with some promise in potential clinical translation.

## Gallium-68 Pet Imaging - Not So New Kid on the Block

The advent of the radioisotope Gallium-68 (^68^Ga) has revolutionised molecular imaging owing to the favourable physical properties of the radioisotope. Gallium-68 is produced from a Germanium-68/Gallium-68 generator (with the parent isotope having a half-life of 270.8 days) and allows for its use for up to 1 year (106). The half-life of ^68^Ga (68 min) mirrors the pharmacokinetics of many well-known peptides and molecules owing to their fast clearance from the blood its biggest impact thus far has been in the imaging of neuroendocrine tumours (107) (when radiolabelled to a somatostatin analogue) and recently with the ligand prostate-specific membrane antigen (PSMA) (108).

The growing interest in infection imaging with ^68^Ga has grown considerable over the last decade (109). A growing number of radiopharmaceuticals chelated to ^68^Ga have been studied within both clinical and pre-clinical settings (refer to Table 2).

**Table 2:** Infection imaging with Gallium-68-labelled compounds.

**Year**	**Authors**	**Journal**	**Tracer**	**Target**	**Application**	**Sensitivity and specificity (if clinical data available)**	
**Pre-clinical**							
2014	Ebenhan et al. (110)	Nuc Med Bio	[^68^Ga]Ga-NOTA-UBI29-41	Bacterial cell wall	TB; Musculoskeletal infections vs. TB		
2015	Mokaleng et al. (111)	BioMed Research Int	[^68^Ga]Ga-DOTA—TBA101	Bacterial cell wall	TB; *E. coli*		
2017	Ebenhan et al. (112)	Molecules	[^68^Ga]Ga-DOTA—TBA101	Bacterial cell wall	*S. aureus* vs. sterile inflammation; TB vs. sterile muscular inflammation		
**Clinical**							
2014	Vorster et al. (113)	Ann Nucl Med	[^68^Ga]Ga-citrate	Transferrin receptor, ferritin, lactoferrin	TB	100	57.7
2016	Vorster et al. (109)	Semin Nucl Med	[^68^Ga]Ga-NOTA-UBI29-41	Bacterial cell wall	Pulmonary TB		
2016	Vorster et al. (109)	Semin Nucl Med	[^68^Ga]Ga-NOTA-RGD	α_v_ β_3_ integrin	TB		
2016	Kang et al. (114)	Semin Nucl Med	[^68^Ga]Ga-alfatide II	α_v_ β_3_ integrin	TB vs. NSCLC	85.71	84.62
2016	Pyka et al. (115)	J Nucl Med	[^68^Ga]Ga-PSMA	PSMA receptor	Prostate cancer. Incidental TB		
2017	Ahuja et al. (116)	Clin Nucl Med	[^68^Ga]Ga-PSMA	PSMA receptor	Prostate cancer, Incidental TB calvarium and lung		
2019	Vorster et al. (117)	Q J Nucl Med Mol Imaging	[^68^Ga]Ga-citrate	Transferrin receptor, ferritin, lactoferrin	TB		
2019	Ankrah et al. (118)	Nuklearmedizin	[^68^Ga]Ga-citrate	Transferrin receptor, ferritin, lactoferrin	TB		
2020	Gupta et al. (119)	Indian J Nucl Med	[^68^Ga]Ga-PSMA	PSMA receptor	Prostate cancer. Incidental spinal TB		
2020	Wong et al. (120)	Clin Nucl Med	[^68^Ga]Ga-PSMA	PSMA receptor	Prostate cancer. Incidental intracerebral TB		
2020	Naftalin et al. (121)	Sci Rep	[^68^Ga]Ga-DOTANOC	Somatostatin Receptor Type 2a	Pulmonary TB		
2020	Gu et al. (122)	Clin Nucl Med	[^68^Ga]Ga-FAPI	Fibroblast-activated protein	Malignancy. Incidental TB lymphadenitis		
2021	Hao et al. (123)	Eur J Nucl Med Mol Imaging	[^68^Ga]Ga-FAPI	Fibroblast- activated protein	TB		

## Pre-Clinical Work With Gallium-68

Agents targeting the *MTb* cell wall were developed by Ebenhan et al. to detect inflammation. The depsipeptide NH2-l-proline-l-Leucine-l Proline-l-Valine-L-leucine-l-Thronine-l-Isoleucine-OHH(TBIA101) when chelated to 1,4,7,10-tetraazacyclododecane-1,4,7,10-tetraacetic acid (DOTA) showed uptake of [^68^Ga]Ga-DOTA-TBA101 in *MTb*-infected rabbits. It was, however, not able to distinguish sterile inflammation from infection (111, 112).

Hypoxia imaging has also played a role in mapping out the pathophysiology of TB granulomas (124). Hypoxia plays a fundamental role in switching metabolism in *MTb* to an inactive state which promotes non-replicating persistent bacteria (125). Imaging the microtumour environment in cancer using radiolabelled hypoxia agents has been established and as such can potentially play a role in managing *MTb* (126). Fluorine-18 fluoromisonidazole ([^18^F]FMISO) demonstrated severe hypoxia in tuberculous lesions in five patients as a primer for clinical translation (62). This is awaiting further clinical translation with a novel Gallium-68-based hypoxia agent to characterise the granuloma environment in TB (125).

The natural antimicrobial agent ubiquicidin (UBI) has been studied over the last decade as an attractive infection imaging agent both with SPECT and with PET agents (127). UBI is a 51 amino acid derivative that has an affinity for targeting bacterial cell wall synthesis. Preliminary results in the pre-clinical setting have shown bacteria-specific binding in rabbits infected with *Staphylococcus aureus* when [^68^Ga]Ga-NOTA-UBI30-41 was utilised (110). ^68^Ga-1,4,7,10-tetraazacyclododecane-1,4,7,10-tetraacetic acid (DOTA)- labelled peptides have also been utilised in infection and inflammation imaging (128). [^68^Ga]Ga-NOTA-UBI has been utilised in the clinical setting demonstrating moderate uptake in a patient with active pulmonary TB (109). However, this should be reviewed to further validate this agent (109, 129).

## Clinical Translation Of Gallium-68-Labelled Agents

Gallium-68 citrate ([^68^Ga]Ga-citrate) has been used as a utility for assessing lesion activity, especially in staging a patient with proven TB. It localises to inflammatory foci by non-specific and specific transferrin dependent and independent mechanisms which are like [^67^Ga]Ga-citrate (129). Preliminary work in a pilot study performed in Pretoria, South Africa reviewed [^68^Ga]Ga-citrate for diagnosis of indeterminate lung lesions (113). Of the patients who were proven to have TB, the work found that tuberculous lesions visible on CT had significant uptake with other lesions (e.g., lung cancer and other benign lesions) having less significant uptake. A follow-up study (5 years later) focusing on patients with proven TB demonstrated uptake in both pulmonary and extrapulmonary TB lesions (117). When compared directly with 2-[^18^F]FDG PET/CT in patients with TB, 2-[^18^F]FDG detected more lesions than [^68^Ga]Ga-citrate. However, [^68^Ga]Ga-citrate had better detection in intracranial TB and is a better agent to use in this context owing to the biodistribution of [^68^Ga]Ga-citrate (118). Intracranial TB has also had limited utility using Gallium-68-NOTA-arginine-glycine-aspartic acid (RGD) ([^68^Ga]Ga-NOTA- RGD) as a potential agent (109).

[^68^Ga]Ga-alfatide II is a marker of angiogenesis. In TB lesions, it has been shown that there is a decrease in the microvessel density from the edge of the granuloma to the central avascular region. Within malignant lesions, the expression of angiogenesis will be much higher. It is this difference that was exploited by Kang et al. to compare to the difference of 2-[^18^F]FDG and [^68^Ga]Ga-alfatide II in differentiating TB and non-small cell lung cancer (NSCLC) (114). The uptake of [^68^Ga]Ga-alfatide II in NSCLC lesions was significantly higher than in TB lesions, hence [^68^Ga]Ga-alfatide II was able to distinguish NSCLC from TB when compared to 2-[^18^F]FDG using angiogenesis as a model.

Gallium-68 PSMA ([^68^Ga]Ga-PSMA) has had much traction recently in its utility in primary staging of high-grade prostate cancer, detection of biochemical recurrence, planning patients for radioligand therapy (130, 131). PSMA is a type II transmembrane glycoprotein that is overexpressed in prostate cancer (132). [^68^Ga]Ga-PSMA has shown many other pathologies which may display uptake of PSMA, particularly in some tuberculous lesions (116).

Pyka et al. in their series of patients showed uptake in two tuberculous lesions, albeit not to be to the same intensity as the prostatic lesions (115). Several case reports have seen PSMA uptake in tuberculous extrapulmonary lesions such as the spine (119), skull (116), and brain (120). No work has been shown in its utility in the management of TB but should be considered as an important pitfall, particularly in TB endemic areas.

Gallium-68 labelled somatostatin receptor imaging (labelled as [^68^Ga]Ga-DOTANOC), [which is a target for somatostatin type 2 receptors and is overexpressed by activated macrophages (133)] has been studied in conjunction with 2-[^18^F]FDG to characterise lesions in TB. The authors concluded that [^68^Ga]Ga-DOTANOC can detect pulmonary TB lesions, but that 2-[^18^F]FDG is still more sensitive for both active and subclinical lesions (121).

Fibroblast-activated protein (FAP) is highly expressed within the stroma of a variety of tumours. Gallium-68 labelled FAP inhibitor (FAPI) has demonstrated its convincing utility in several oncologic applications (134–136), with advantages being no major patient preparation and improved better target to background ratio in some cases. There are a few cases using [^68^Ga]Ga-FAPI in incidental detection of TB in lymph nodes (122) and intracerebral TB (123) and may bear some promise as the tracer develops further into evaluating other non-oncologic applications.

## The Fundamental Problem of The Tuberculous Granuloma

One of the key problems in the pathogenesis of TB is the development of the granuloma, which is the pathological hallmark of this disease (137, 138). Heterogenous forms exist with a spectrum of host outcomes (137, 139, 140). Transmissive granulomas harbour an abundance of neutrophils which will either protect the host by driving cell priming and granuloma formation or promote disease severity (141). Neutrophils recognise *MTb* by releasing lysosomal enzymes, human neutrophil peptides, and a host of other reactive oxygen species which aim to lyse the mycobacterium. A second mechanism exists where neutrophils release neutrophil extracellular traps which aim to trap the microbe and thereby present its further action on the host (142, 143).

It has been shown that neutrophilia portends a poorer prognosis and independently predicts death in patients with TB (144). The ability to image the neutrophil and its role in TB pathogenesis will assist in identifying factors promoting immunopathology, which may be targeted for the development of specific host-directed therapies that will add to the current armamentarium of diagnostic and therapeutic options available for TB. Neutrophils may be found in patients with LTBI and may have a role in identifying those patients that may be at risk of developing the active disease (145). With 2-[^18^F]FDG being a glucose analogue, its mechanism of uptake [which is driven by glycolytic metabolism for generation of adenosine triphosphate (ATP)] does not allow for differentiation between different immune cell populations (40, 98, 146). This is despite multiple studies reviewing the utility of quantitative measures to be able to predict response to therapy in patients with pulmonary and extrapulmonary TB (147–151) (including dual-time-point imaging) (44).

## Introducing the Formyl Peptide Receptor

Leucocytes will accumulate at sites of inflammation by responding to pathogen and host-derived factors known as chemo attractants. The formyl peptide receptor (FPR), a G protein-coupled receptor, has the ability to recognise the N formyl methionine motif in synthetic neutrophil chemotactic peptides (152). It is a classic G protein-coupled receptor with seven transmembrane-spanning regions with an N terminus (extracellular) and C terminus (intracellular), and three loops exposed on the cell surface for ligand interaction (153).

FPR1, in particular, is expressed by neutrophils, which play a fundamental role in chemotaxis, killing microorganisms through phagocytosis, and the generation of reactive oxygen species (154, 155). These peptides were first seen to be an attractant for neutrophils in early pre-clinical work (156–158).

As a result of early work performed using the *Escherichia coli*-derived synthetic ligand formyl-methyl-leucine-phenylalanine (fMLF), much is now known on the properties of FPR1. Once FPR1 is activated, fMLF is internalised within 30 seconds and then leads to a cascade of events which include transcription regulation, superoxide production, degranulation, cytokine expression, and changes in cell surface marker expression (159).

## A Brief History of The Fpr1 Molecular Probe

In the early 1990s, work was performed with ^99m^Tc-labelled chemotactic peptide receptor antagonist [N-fMLF-lysine(N-For-MLK)] with high biological retention and receptor-binding activity in sites of infection in rats and rabbits (160–162). The tracer also showed a high target to background ratio seen when compared to radiolabelled leucocytes (163) or human polyclonal immunoglobulin G (164). The problem with this agent was the poor image quality owing to its high lipophilicity which resulted in higher liver uptake (161). This peptide [N-cinnamoyl –F-(D) L – F – (D) –L F (cFLFLF), an antagonist for FPR1], has been explored with a new agent polyethylene glycol (PEG) to decrease the lipophilicity and mitigate liver uptake and aimed to radiolabel with Copper 64(^64^Cu). Copper 64 has a half-life of 12.7 h and a unique decay profile which makes it attractive for radiolabelling with various peptides and molecules for PET/CT imaging and potential radionuclide therapy (with emission of Auger electrons and β-particles) (165, 166). In the mouse model, this was able to detect lung inflammation (167). This was confirmed two years later where [^64^Cu]Cu- (PEG)-cFLFLFK has been shown to bind to FPR1, accumulating at sites of inflammation *in vivo*, with LE of more than 95%. Work with this tracer in mice infected with *Klebsiella* confirmed that the cellular infiltrates in the lungs was exclusively neutrophils at the time of imaging (confirmed with immunohistochemistry) (168). This has also been confirmed with another radiolabelled tracer, [^68^Ga]Ga-nanotracer-cFLFLF, in the murine model with clinically proven relevant neutrophil recruitment to sites of infection and LE >97% (169).

## Pre-Clinical Technetium 99M-Based Applications

^99m^Tc-labelled-cFLFLF was considered owing to the ready availability of ^99m^Tc from its parent isotope, molybdenum-99. [^99m^Tc]Tc-PEG12-TKPPR-cFLFLF had a high affinity for neutrophils and was stable in serum with sufficient hydrolipophilicity (170). The probe showed feasibility for *in vivo* imaging of acute neutrophilic inflammation in the murine model. Stasiuk et al. also demonstrated the feasibility of [^99m^Tc]Tc-cFLFLFK-NH_2_ in the murine model to show inflammation non-invasively (171–173).

Chen et al. reviewed using [^99m^Tc]Tc-cFLFLF in rats with acute osteomyelitis (AO). This group used both methylene diphosphonate (MDP) (a bone-seeking agent with clinical utility in osteomyelitis) (86) and 2-[^18^F]FDG (87, 174) to ascertain early diagnosis and therapeutic monitoring of AO. The work aimed to investigate [^99m^Tc]Tc-cFLFLF in the rat model of AO and compare it with conventional imaging with [^99m^Tc]Tc-MDP bone scan and 2-[^18^F]FDG. The group had 40 rats which were divided into eight groups (A–H) of five each. Three of these groups were designated as sham controls (D, E, and F) and the remainder were the AO models. Groups A and D had [^99m^Tc]Tc-cFLFLF scintigraphy, Groups B and E had [^99m^Tc]Tc-MDP, and Groups C and F had 2-[^18^F]FDG. [^99m^Tc]Tc-cFLFLF was superior to [^99m^Tc]Tc-MDP and 2-[^18^F]FDG in the detection of acute AO with a high affinity for neutrophil binding with FPR1. In addition, the probe was able to monitor the effects of therapy for AO, highlighting the ability of [^99m^Tc]Tc-cFLFLF in diagnosis and monitoring of therapeutic interventions (175).

Another group aimed to exploit the pathophysiology of acute intervertebral disk herniation by tracking leukocyte filtration using FPR1-mediated molecular imaging. Using SPECT and near-infrared fluorescence imaging (NIRF), the probe [^99m^Tc]Tc-HYNIC-PEG-cFLFLF was injected into sham- and disc-injured mice. Neutrophils were detected from days 1 to 3 with the preferential accumulation of [^99m^Tc]Tc-HYNIC-PEG-cFLFLF in herniation sites, hence demonstrating directly and non-invasively the utility of FPR1-targeted molecular imaging in tracking inflammatory processes (176).

FPR1-mediated imaging has a role in the non-invasive diagnosis of lung ischaemic reperfusion injury post-lung transplantation which can lead to primary graft dysfunction (PGD) as the main morbidity (177). Lung inflammation was seen on SPECT imaging in mice with [^99m^Tc]Tc-cFLFLF at 2, 12, and 24 h post-reperfusion. A porcine model was used to confirm its translational aspect. This may have implications in the early diagnosis of PGD which impacts therapeutic interventions and outcomes of patients (178, 179).

In another study mapping the pathogenesis of abdominal aortic aneurysms (AAA), [^99m^Tc]Tc-cFLFLF was used to track the role of neutrophil infiltration and activation in the evolution of AAA. In the murine model, an elastase treatment model of AAA was developed. [^99m^Tc]Tc-cFLFLF was observed to accumulate in aortic tissue treated with elastase when compared to the controls. A marked increase in neutrophil accumulation was observed in aortic tissue, confirming the role of non-invasive molecular imaging to help map out pathophysiology using [^99m^Tc]Tc-cFLFLF (180).

## Translation Into Work With TB

More recent work with TB granulomas using human neutrophils and a mouse model was performed to evaluate the *in vivo* cellular specificity of cFLFLFK-PEG12-Cyanine 3(Cy3) within a human *in vitro* granuloma model and a mouse model of lung granulomatous inflammation (181). The human neutrophils were taken from patients who had LTBI. The probe used in the work was found to preferentially bind to neutrophils than to monocytes or lymphocytes in human cells. In the mouse model, cFLFLFK was found to be accumulating more within the granulomatous inflammatory responses in the lung, preferentially localised to the neutrophils and cells of the monocyte or macrophage lineage (181). This has set the ground for aiming to understand granuloma biology and the role of cells expressing FPR1 in contributing to TB pathogenesis. In the macaque model, cFLFLF peptide binds specifically to neutrophils in inflammatory cells in the mouse model, to neutrophils in macaque blood, and neutrophils in the granulomas from the necropsy. It bound poorly to macrophages and lymphocytes (41). This was further translated recently in comparison with 2-[^18^F]F-FDG where [^64^Cu]Cu-cFLFLF was retained in lung granulomas in *MTb*-infected cynomolgus macaques. There was a positive correlation with neutrophils and macrophages (and to a lesser extent T and B cells) when the granulomas were further analysed (42).

## Future Perspectives

The neutrophil is known to be part of both host protection and inflammation processes causing damage in TB. The abundance of neutrophils within a granuloma is associated with a poorer prognosis and mortality in TB. In patients with TB granulomas, the presence may result in progression to active TB from an LTBI. This is particularly relevant in lesions where there is a high mycobacterial load or some form of immune dysfunction. These are inherently dependent on the host immunity, stage of disease, and virulence of the mycobacterium itself (144, 182).

Molecular imaging, particularly FPR1-mediated molecular imaging, has great potential in being able to assist in the management and therapy of a wide variety of pathological diseases and processes.

**Figure 3:**
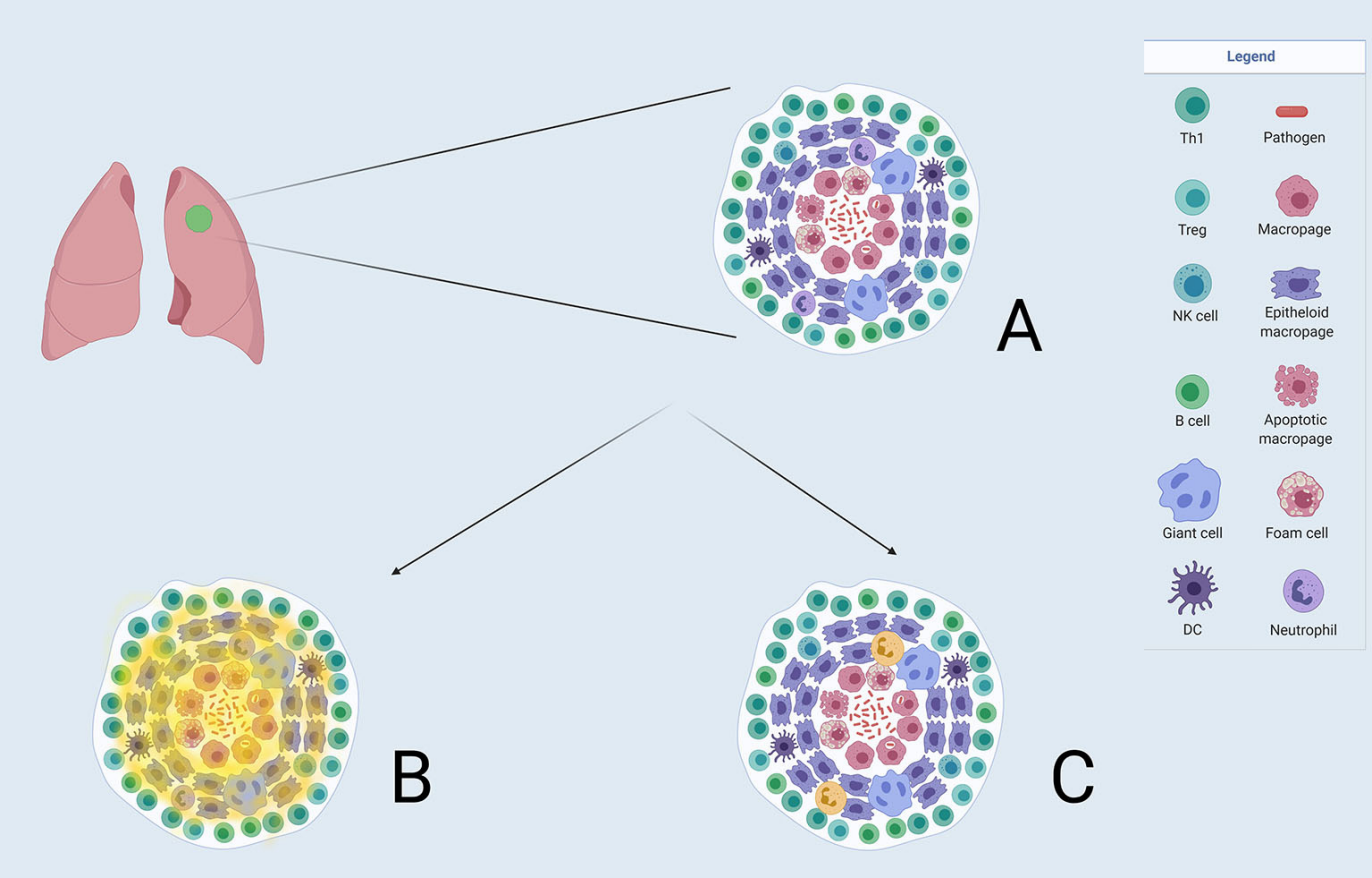
**(A)** A TB granuloma with the constellation of different cell types. Macrophages are seen at the centre which can be differentiated into other cell types illustrated. Neutrophils form part of the granuloma. **(B)** Demonstrates where the 2-[^18^F]FDG uptake(in faint yellow) is distributed in a granuloma, and not being able to discriminate between the relevant cell types. **(C)** Shows the projected uptake of FPR1-mediated molecular imaging (in bold yellow) with Gallium-68-nanotracer-cFLFLF highlighting neutrophils in the granuloma.

The utilisation of these novel probes will be able to characterise transmissive granulomas from those that are protective (refer to Figure 3). This could enable the identification of patients who may be at risk of relapsing at the end of their TB therapy and possibly, more importantly, may have a role in identifying those patients with LTBI that may be at risk of developing active disease. As the tracer [^64^Cu]Cu-cFLFLF in conjunction with 2-[^18^F]F-FDG has recently been proven utility in the diagnosis and monitoring of TB in cynomolgus macaques (42), it is the hope that this and future work can be translated adequately into the clinical spectrum which will assist in the resolution of many diagnostic dilemmas and challenges within the TB spectrum and thereby make this tracer a suitable option for routine clinical work.

## Author Contributions

SM wrote the manuscript. All authors planned the manuscript content, analysed the literature, wrote parts of it, and edited the final manuscript.

## Funding

The authors acknowledge funding from the Wellcome Trust (MM, 206751/Z/17/Z), Bill and Melinda Gates Foundation (MM), and (OPP1210776) SA Medical Research Council (SAMRC) with funding from the SA Department of Health (MM).

## Conflict of Interest

The authors declare that the research was conducted in the absence of any commercial or financial relationships that could be construed as a potential conflict of interest.

## Publisher's Note

All claims expressed in this article are solely those of the authors and do not necessarily represent those of their affiliated organizations, or those of the publisher, the editors and the reviewers. Any product that may be evaluated in this article, or claim that may be made by its manufacturer, is not guaranteed or endorsed by the publisher.
